# Efficient and Cost Effective Population Resequencing by Pooling and In-Solution Hybridization

**DOI:** 10.1371/journal.pone.0018353

**Published:** 2011-03-30

**Authors:** Vikas Bansal, Ryan Tewhey, Emily M. LeProust, Nicholas J. Schork

**Affiliations:** 1 Scripps Genomic Medicine, Scripps Translational Science Institute, La Jolla, California, United States of America; 2 Division of Biological Sciences, University of California San Diego, La Jolla, California, United States of America; 3 Genomics, Agilent Technologies, LLSU, Santa Clara, California, United States of America; 4 Department of Molecular and Experimental Medicine, The Scripps Research Institute, La Jolla, California, United States of America; Duke-National University of Singapore Graduate Medical School, Singapore

## Abstract

High-throughput sequencing of targeted genomic loci in large populations is an effective approach for evaluating the contribution of rare variants to disease risk. We evaluated the feasibility of using in-solution hybridization-based target capture on pooled DNA samples to enable cost-efficient population sequencing studies. For this, we performed pooled sequencing of 100 HapMap samples across ∼600 kb of DNA sequence using the Illumina GAIIx. Using our accurate variant calling method for pooled sequence data, we were able to not only identify single nucleotide variants with a low false discovery rate (<1%) but also accurately detect short insertion/deletion variants. In addition, with sufficient coverage per individual in each pool (30-fold) we detected 97.2% of the total variants and 93.6% of variants below 5% in frequency. Finally, allele frequencies for single nucleotide variants (SNVs) estimated from the pooled data and the HapMap genotype data were tightly correlated (correlation coefficient > =  0.995).

## Introduction

Over the past few years, genome wide association studies (GWAS) have uncovered hundreds of common variants associated with various traits and common diseases[1]. The discovery of these disease-associated variants has shed light on previously unknown genes and disease mechanisms. However, for many diseases, a large proportion of the genetic variation underlying disease risk remains to be discovered. There is growing evidence that some of this ‘missing heritability’ could be explained by the influence of collections of rare variants (MAF <0.05) which have not been captured by current genotyping chips used in most GWAS[2], [3].

The contribution of rare variants to phenotypic variation can be effectively surveyed through large-scale population re-sequencing studies. Due to high costs, most re-sequencing studies have been limited to sequencing the coding regions of a small number of genes using Sanger sequencing[4]. The availability of high-throughput target capture methods combined with the massive throughput of next-generation sequencing platforms has made it possible to interrogate thousands of genomic loci in a cost effective manner[5], [6]. In fact, the costs are so low that when one factors only the price to run the sequencer and the production of raw base reads, it is feasible to sequence megabases of DNA in thousands of individuals using a small number of sequencing runs. The budgetary bottleneck for such studies is not the sequencing cost but the cost of sample preparation for each individual sample prior to sequencing. Assuming that it costs ∼250 dollars for target capture and library preparation, the sample preparation cost for a project with 1000 individuals would be $250,000. A simple strategy to reduce the per-sample cost while utilizing the massive capacity of current sequencers is to use DNA pooling prior to target capture and sample preparation. DNA pooling was previously proposed as a strategy for reducing the cost of large-scale genotyping-based disease association studies[7]. However, the difficulty in accurately measuring allele frequencies from intensity data has limited the use of this strategy. Unlike pooled genotyping, pooled DNA sequencing not only provides digital allele counts for each variant but can also be used to detect novel sequence variants. Several recent studies have demonstrated the potential of pooled sequencing using next-generation sequencing platforms for identifying disease associated rare mutations [8], [9].

There are two main requirements for the successful utilization of DNA pooling for large-scale resequencing studies. Prior to sequencing, it is important to minimize various biases that cause unequal representation of DNA from individuals in the pooled sequencing library. Such pooling imbalances can result in inaccurate estimates of allele frequencies as well as make it difficult to identify rare variants. Post-sequencing, it is essential to identify rare and common variants with low false positive and false negative rates. Detection of rare variants from pooled sequencing data represents a particularly challenging task since the signal for rare variants can be difficult to distinguish from sequencing errors[10].

While careful quantification of genomic DNA samples prior to pooling is necessary to reduce imbalances, biases introduced in the target-capture step need to be minimized as well. In comparison to PCR-based enrichment strategies which are sensitive to DNA quality and can introduce biases[11], in-solution sequence hybridization is less likely to introduce imbalances in the pooled DNA sequencing library. In this study, we evaluated the feasibility of in-solution sequence hybridization-based enrichment of genomic loci applied to pooled genomic DNA. We sequenced ∼600 kilobases of coding sequence across the human genome in 100 HapMap samples using 5 pools with 20 samples each. We used Agilent in-solution enrichment for target enrichment and sequenced the pooled libraries using the Illumina Genome Analyzer. Using a statistical variant caller for pooled sequencing data, CRISP[12], we were able to detect single nucleotide variants with high sensitivity and specificity. Comparison of the pooled allele frequency estimates to data from the HapMap and 1000 Genomes project demonstrated the ability to accurately estimate allele frequencies from pooled sequencing. In addition, we were also able to accurately identify short insertion/deletion variants from the pooled data.

## Results

### Study design and sequencing

We targeted 594 Kb of DNA sequence from the coding regions of genes that were also sequenced in the 1000 Genomes exon pilot project[12] by solution based hybridization capture (Table S2). For sequencing, we selected 100 HapMap samples: 20 Utah residents with ancestry from northern and western Europe (CEU), 20 Han Chinese in Beijing, China (CHB), 20 Tuscans in Italy (TSI) and 40 Yoruba in Ibadan, Nigeria (YRI)[13] (Table S1). The samples were pooled by population with each pool consisting of 20 individuals. We chose a moderate pool size of 20 so that the allele frequency of a singleton variant (1/40) was well above the average sequencing error rate of an Illumina GAIIx, which we observe to be between 0.5–1%. Pooled samples were carried through the library preparation process as well as the sequence enrichment process following the normal protocol, as though they were from a single genomic sample. The 5 pools were sequenced using DNA barcodes on two lanes of the Illumina GAIIx using 55 bp paired-end reads.

### Accuracy of SNV detection and allele frequency estimates

Reads for each pool were aligned to the human genome reference sequence.

(HG18) using the BWA aligner[14]. We generated an average of 695 Mb of sequence data for each pool, ∼55% of which mapped directly on our 594 Kb of target sequence (Table 1). On average each pool had 683-fold coverage across the targets, translating to an average of 34-fold coverage per individual. For calling variants, we utilized a statistical method, CRISP, that is designed for variant detection using sequence data from multiple DNA pools[15]. Within the targeted regions, we detected 2849 variants (2749 SNVs and 100 indels) using this method.

**Table 1:** Capture Efficiency.

	Total	On Target	Mean	% of Bases
Pool	Sequence	Sequence	Coverage	Between 1/5 and 5x
				Mean Coverage
CEU	722 Mb	401.8 Mb (55.7%)	676	95.7%
CHB	709.6 Mb	388.7 Mb (54.8%)	654	94%
TSI	694.7 Mb	376.8 Mb (54.2%)	634	94.1%
YRI 1	606.4 Mb	327.8 Mb (54.1%)	552	92.6%
YRI 2	744.6 Mb	415.2 Mb (55.8%)	699	94.6%

To assess the accuracy of the SNV calls and the allele frequency estimates, we generated a merged set of variants and genotypes using data on the same set of 100 samples from the HapMap and the 1000 Genomes projects (HM+1KG dataset). Further, we restricted the analysis to the 470 kb of sequence that overlapped with the exon capture boundaries of the 1000 Genomes pilot project. We compared the SNVs in each pool to the SNVs reported in the HM+1KG dataset for the corresponding 20 samples in the pool. Summed across the 5 pools, we identified 4588 SNVs, 4327 of which matched the HM+1KG dataset (Table 2). Across the 470 kb of sequence, we failed to detect 490 SNVs called in the HM+1KG dataset. Many of these missed variants are likely due to inadequate sequencing depth in our data. Indeed, false negative rates reduced to 6% (276 variants) and 2.8% (82 variants) when considering bases with at least 10 and 30-fold average coverage per sample respectively (Table 2). Not surprisingly, the majority of these variants were of low frequency with 266 of the variants at 10-fold coverage and 81 of the variants at 30-fold coverage being at an allele frequency of 5% or lower. This represents a sensitivity of detection of 86.3% at 10-fold and 93.4% at 30-fold coverage per individual for variants present on 2 or fewer chromosomes in the pool. Next, we assessed the false discovery rate of our SNV calls. Because of missing data in the 1000 genomes dataset for three of the pools, we only used the TSI pool and one of the YRI pools for this analysis. Taking into account all bases in the two pools, we identified 112 SNVs not reported in the HM+1KG dataset. Of these, 42 (37.5%) were previously reported in dbSNP (v130) lending support for them to be true variants. The majority (74.1%) of the false positives were of low frequency (< = 5%). Of the variants estimated to be of 5% frequency or lower in our data, 23 were present in dbSNP. For the remaining 70 sites, we visually inspected aligned sequence reads from the 1000 Genomes project to evaluate if they represented potentially real variants. 49 of the 70 sites appeared to be true polymorphisms, 8 sites had little or no coverage for one or more samples. The remaining 13 sites were clearly false positives in our dataset, 6 of the 13 sites had an estimated allele frequency of 5% or lower. Assuming that half of the low coverage sites represent true variants, we estimate a false discovery rate of 0.9% for our pooled variant calls.

**Table 2:** Variant Detection Statistics for Pooled Sequencing.

			Coverage > = 10^1^					Coverage > = 30^1^				
	Total Variants	Detected	Detected	False Positives^2^				Detected	False Positives^2^			
Pool	HM & 1KG	Variants	Variants	in dbSNP 130		False Negative^3^		Variants	in dbSNP 130		False Negative^3^	
	at Targets	in Pool	in Pool	Absent	Included				Absent	Included		
CEU	826 (283)	744 (213)	702 (213)	22 (15)	11 (6)	47 (45)	6.4% (19.2%)	479 (158)	16 (12)	10 (6)	17 (17)	3.4% (9.7%)
CHB	731 (268)	645 (194)	599 (191)	34 (29)	9 (7)	56 (56)	8.7% (24.7%)	410 (150)	25 (22)	8 (6)	22 (22)	5% (12.4%)
TSI	850 (377)	748 (287)	700 (280)	37 (33)	26 (15)	54 (53)	7.1% (16.2%)	460 (198)	29 (26)	24 (13)	10 (10)	1.9% (4.2%)
YRI 1	1194 (419)	1084 (338)	995 (324)	56 (50)	13 (6)	54 (50)	5.1% (13.2%)	551 (192)	31 (29)	11 (5)	17 (16)	2.9% (7.1%)
YRI 2	1216 (573)	1106 (482)	1041 (470)	33 (27)	15 (8)	65 (62)	6% (12.3%)	712 (332)	23 (19)	15 (8)	16 (16)	2.1% (4.5%)
Sum	4817 (1920)	4327 (1514)	4037 (1478)	182 (154)	74 (42)	276 (266)	6% (13.7%)	2612 (1030)	124 (108)	68 (38)	82 (81)	2.8% (6.4%)

The number in parentheses represents only variants at 5% or lower frequency in the dataset.

1- Statistics for variant sites which were sequenced to a depth of 10 or 30 fold per individual in the pooled dataset.

2- Variants called in the pooled dataset not present in either HapMap or the 1000 Genome Project. Variants were further classified as being included or absent in dbSNP v130.

3- Variants called in the HapMap or 1000 Genome Project that were not called in our pooled dataset.

**Figure 1:**
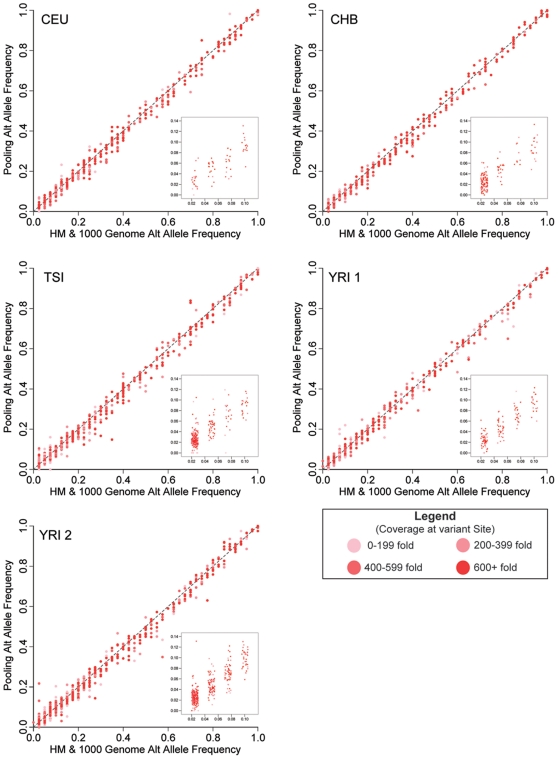
Comparison of pooled allele frequency estimates with actual allele frequencies. Scatter plots for each of the 5 pools (CEU, TSI, CHB, YRI 1, YRI 2) of the estimated allele frequency as calculated by read counts from the sequence data plotted against the actual allele frequency from either the HapMap or 1000 Genome project. Only sites that contained genotype information for all 20 individuals in that particular pool are included. The insert displays the area of the graph representing 1–3 copies of the alternate allele as a jitter plot. In both graphs, the points are shaded to represent overall read coverage in our sequencing data at that site.

**Figure 2:**
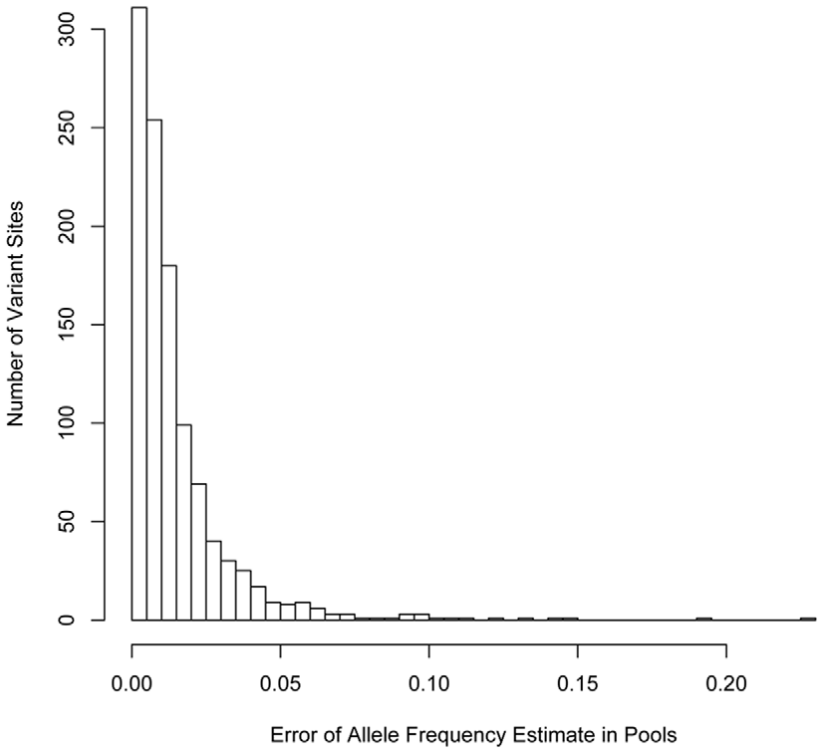
Error in the pooled allele frequency estimate for each variant. Histogram of the estimated error in measurement of allele frequency from the pooled sequencing data. For each variant, the absolute difference between the pooled allele frequency estimate and the actual allele frequency derived from the 1000 Genomes or HapMap data was computed.

We then compared the allele frequencies calculated using the read counts in the pools to the actual allele frequencies determined from the HM+1KG genotypes for each pool. The correlation between the HM+1KG allele frequencies and the pooled estimates was excellent with an r^2^ correlation coefficient ranging from 0.995–0.997 across the five pools and very few outliers (Figure 1). In addition, our estimated allele frequencies for the vast majority of variants closely matched those from the HM+1KG with 80% of the variants within 1/40^th^ and 94% within 1/20^th^ of the actual frequency (Figure 2).

### Detection of indel variants

The ability to detect short insertion/deletion variants from pooled sequence data is useful, especially in coding regions of the genome. Detection of short indels from next-generation sequencing data is challenging due to the difficulty in accurately aligning short reads with gaps and the lack of an indel error model for short reads. The statistical model underlying our variant caller, CRISP, uses allele counts across multiple pools to differentiate variants from sequencing errors and is also applicable to indels. CRISP identified 100 indels (1–18 bp) in the 5 pools across the 570 kb of target sequence. Only 19 indels were called in the 1000 genomes exon project and of these 5 overlapped our target sequence. 4 of these 5 indels matched indels called in our pooled data. An additional 55 indels were identical to indels reported in dbSNP (v130) or in the 1000 Genomes whole-genome low coverage variant calls[13]. To validate the remaining indels, most of which were low frequency variants, we visually examined the aligned sequence reads for the 100 samples sequenced in the 1000 Genomes Project (see Materials and Methods) and found clear evidence for 28 indels. Overall, 87 of the 100 pooled indel calls were validated.

### Cost Efficiency and power of Pooled Sequencing

We sought to compare the costs of a resequencing project between pooled and individual sequencing using in-solution hybridization. We evaluated two scenarios, a small and large capture consisting of 750 kb and 3 Mb of total targeted regions respectively on a small (400), modest (4,000) and large (20,000) cohort of samples. The cost savings afforded by fewer library preps represented an 11.8-fold decrease with the 750 kb capture and a 5.7 fold decrease for the 3 Mb capture when sequencing to 20x coverage per sample (Table 3). The tight correlation between the pooled allele frequency estimates and the individual-based allele frequencies in our study shows that the increase in the variance of the allele frequency due to the pooling noise is small. The ability to sequence more samples can offset this slight increase in the variance of the allele frequencies and substantially enhance the power to detect associations across a range of minor allele frequencies and effect sizes [16], [17].

**Table 3:** Cost Estimates for Pooled Sequencing Projects.

			750 Kb Capture Project				3 Mb Capture Project			
	Library Prep			Total Project				Total Project		
	Cost ($1000)		Sequencing	Cost ($1000)		Cost	Sequencing	Cost ($1000)		Cost
Number of			Cost ($1000)			Difference	Cost ($1000)			Difference
Samples	Single Plex	Pooled		Single Plex	Pooled			Single Plex	Pooled	
400	110	5.5	2.7	112.7	8.2		10.7	120.7	16.2	
4000	1100	55	26.8	1126.8	81.8	13.8	107	1207	162	7.5
10000	2750	137.5	66.9	2816.9	204.4		267.6	3017.6	405.1	

Cost estimates based on a 100 bp HiSeq paired-end run with Illumina's published reagent costs ($11,150 per flowcell) and an average throughput of 200 Gb per run. Sample preparation includes $75 per samples for library prep and $200 per sample for solution based capture. All calculations for pooled sequencing assume 20 individuals per pool.

## Discussion

Our results demonstrate that in-solution hybridization capture of pooled DNA samples when combined with our variant calling algorithm is a viable and cost-effective approach for performing large-scale resequencing studies using high-throughput sequencing technologies. Using pooled sequence data from 100 HapMap samples we have shown that this approach minimizes false positives, while being sensitive enough to detect even low frequency alleles, a problem that has plagued previous pooling and sequencing strategies. The ability to accurately detect rare variants including indels from pooled sequence data can enable large-scale sequencing studies of GWAS-associated genes or loci for a particular disease to assess the collective contribution of low-frequency functional variants.

Comparison of the pooled allele frequency estimates with actual allele frequencies derived from HapMap or 1000 Genomes data shows that the pooled allele frequencies are highly accurate. This was clear proof that in-solution target capture introduced virtually no biases in the pooled sequencing library. The ability to accurately estimate allele frequencies from pooled sequencing data is important since it can allow tests for association to be performed directly from the pooled sequencing data without additional genotyping. Furthermore, the ability to sequence a large number of samples via pooling can substantially increase the power to detect common and rare allele associations while still providing a significant reduction in cost compared to individual sequencing. An added benefit of the pooling approach is that only 150 ng of DNA per individual is required which can be important for studies with a limited supply of genomic DNA.

In conclusion, the solution we have presented should prove to be a cost-effective and easy to use approach for researchers looking to perform large targeted re-sequencing studies examining both common and rare variants.

## Materials and Methods

### Sample Preparation and Sequencing

We selected 100 samples (Table S1), 60 of which were from the HapMap phase I+II (CEU, CHB, YRI 1) and the remaining 40 were genotyped as part of HapMap phase II (TSI, YRI 2). All 100 samples were slated for exome capture and sequencing as part of the 1000 Genome Project. DNA was obtained from the Coriell Institute (Coriell Institute); all samples were quantified in quadruplicate using picogreen (Life Technologies). Samples were pooled in equal molar concentrations in pools of twenty samples each. The pools were then carried through the standard Illumina library preparation process using Adaptive Focused Acoustics for shearing (Covaris), end-repair, A-tailing and ligation (New England Biolabs). SureSelect in-solution hybridization was performed on the pooled samples using the recommended protocol for a single genomic DNA sample. Captured DNA was then sequenced using 55 bp PE multiplexed read protocol on an Illumina GAIIx.

### Mapping & Variant Calling

We used the BWA aligner[14] (v 0.5.7) to align the sequencing reads for each pool to the NCBI reference human genome sequence (ncbi36). Short read aligners such as BWA align each read independently to the reference genome and are unable to properly align reads that contain an insertion/deletion event close to the start or end of the sequenced reads. Such misaligned reads can result in false SNVs calls[18], make it difficult to call indels and can be especially problematic for pooled sequencing data. To rectify this problem, we utilized a simple realignment approach in which the reads were aligned (without gaps) to an ‘indel-sensitive’ reference sequence generated from consensus sequences of indels identified from the original BWA alignments. Alignments for reads for which the new alignment, if any, was better than the original BWA alignment were changed. This realignment procedure changed the alignments of a small fraction of reads. The realigned SAM files were used for variant calling using a recently developed statistical algorithm called CRISP that uses information from aligned sequence reads within each pool as well as across all pools. For variant calling using CRISP, reads with low mapping quality (<20) and base calls with a low quality score (<10) were discarded. Variants (SNVs and indels) were identified using CRISP on the aligned sequence reads for the 5 pools using the default thresholds. To call a variant, CRISP required at least 4 reads supporting the non-reference allele in one or more pools. The raw and aligned Illumina reads and the set of variants identified in this study can be downloaded from the following website: http://polymorphism.scripps.edu/datasets/PooledSequencing

### Variant Comparisons

For evaluating variant calls and allele frequencies, we use variant calls and genotypes from the July 2010 release of the 1000 Genome Project's exon sequencing pilot project as well as indel calls from the whole-genome low-pass pilot project[12]. To fill in missing genotypes for some samples, we utilized genotypes from the HapMap public release #28 (Phase I, II+III). Allele frequency comparisons were limited to sites at which there was complete data available for all 20 samples in the corresponding pool. We downloaded aligned sequence reads generated using the Illumina platform for the 100 samples sequenced in our study from the 1000 Genomes ftp website (ftp://ftp-trace.ncbi.nih.gov/1000genomes/ftp/pilot_data/).

For each sample, we generated pileup files and used them for visual confirmation of novel variant calls. All calculations and figures were produced using a combination of Perl, Python and R.

## Supporting Information

Table S1 **A list of the 100 HapMap samples sequenced in this study.**
(XLS)

Table S2 **An Excel file with the list of genomic regions targeted in this study.**
(XLS)
